# Establishment and Verification of Synchronous Metastatic Nomogram for Gastrointestinal Stromal Tumors (GISTs): A Population-Based Analysis

**DOI:** 10.1155/2020/8493707

**Published:** 2020-01-27

**Authors:** Yuqiang Li, Guangfeng Zhang, Xiangping Song, Lilan Zhao, Cenap Güngör, Dan Wang, Wenxue Liu, Yan Huang, Fengbo Tan

**Affiliations:** ^1^Department of Gastrointestinal Surgery, Xiangya Hospital, Central South University, Changsha, China; ^2^Department of General Visceral and Thoracic Surgery, University Medical Center Hamburg-Eppendorf, Hamburg, Germany; ^3^State Key Laboratory of Quality Research in Chinese Medicine, Macau University of Science and Technology, Macao, China; ^4^Department of Rheumatology, Guangdong Provincial People's Hospital, Guangdong Academy of Medical Sciences, Guangzhou, China; ^5^Department of Thoracic Surgery, Fujian Provincial Hospital, Fuzhou, China; ^6^Key Laboratory of Protein Chemistry and Developmental Biology of Ministry of Education, College of Life Sciences, Hunan Normal University, Changsha, China; ^7^Department of Neurosurgery, Second Affiliated Hospital of Hunan Normal University, Changsha, China; ^8^Hunan Provincial Key Laboratory of Neurorestoration, Changsha, China

## Abstract

**Aim:**

Assess the risk of synchronous metastasis and establish a nomogram in patients with GISTs.

**Methods:**

Surveillance, Epidemiology and End Results database (2004-2014) was accessed. With the logistic regression model as the basis, a nomogram was constructed.

**Results:**

7,256 target patients were contained in our study. The nomogram discrimination for mGIST prediction revealed that tumor size contributed most to synchronous metastasis, followed by lymph nodes, extension, pathologic grade, tumor location, and mitotic count. *C*-index values of predictions were 0.821 (95% CI, 0.805-0.836) and 0.815 (95% CI, 0.800-0.831), and Brier score were 0.109 and 0.112 in training and validation group, respectively. The value of area under the ROCs were 0.813 (*p* < 0.001) in the primary cohort and 0.819 (*p* < 0.001) in the validation cohort. Through the calibration curves (as seen in the figures), nomogram prediction proved to have excellent agreement with actual metastatic diseases.

**Conclusion:**

A new nomogram was created that can evaluate synchronous metastatic diseases in patients with GISTs.

## 1. Introduction

Gastrointestinal stromal tumors (GISTs) constitute the most frequent mesenchymal malignancy of the digestive tract, with an approximate incidence of 7.8 cases per million people per year [1]. It is now well accepted that all GISTs can exhibit malignant behavior and none of them can be labeled as definitely benign based on clinicopathologic features. Similar to other malignant tumors, GIST can also be metastasized to other sites of the body [2] and the incidence of metastatic diseases is 15-50% in patients with GISTs [3, 4]. Timely detection of metastatic disease is crucial since metastases are considered to be major factors associated with mortality.

Due to the rareness of GISTs, there is currently no coefficient scoring system to assess the risk of synchronous metastatic GISTs (mGISTs). In fact, identification of risk factors for synchronous metastases can have a great influence on the changes in surveillance and management strategies in GISTs, such as administration of preoperative imatinib and follow-up cycles. Therefore, it is extremely important to create a nomogram that can evaluate synchronous metastatic diseases in patients with GISTs. Moreover, clinical characteristics should be focused since the advantages of convenient availability and wide applicability [5]. The Surveillance, Epidemiology, and End Results database (SEER database) is a kind of population-based cancer registration system of the USA taking 34.6% Americans into account, which can provide some necessary clinical data and be used to be an excellent database to explore GISTs.

Therefore, a nomogram in patients with GISTs was created to assess the synchronous metastatic risk based on clinical factors by analyzing the SEER database in our study.

## 2. Materials and Methods

### 2.1. Patients

Data in this retrospective analysis were extracted from the SEER-linked database. The SEER Program of the National Cancer Institute is an authoritative source of information on cancer incidence and survival in the United States (US) that is updated annually. SEER currently collects and publishes cancer incidence and survival data from population-based cancer registries covering approximately 34.6% of the population from the US [6]. The target population was limited to the patients with GISTs, including GIST esophagus, GIST stomach, GIST small Intestine, GIST peritoneum, GIST appendix, and GIST colorectum, diagnosed in the periods of 2004-2015, 7,635 patients in total. Exclusion criteria are as follows: unknown metastatic status and code of CS tumor size is 0. The final study sample contained 7,256 patients.

For each patient, the following data were acquired: age at diagnosis, gender, race, insurance, tumor size, location, grade, extension, mitotic count, lymph nodes status, and metastatic status. We divided patients into metastatic GISTs (code: 10-60) and nonmetastatic GISTs (code: 0,) according to CS Mets at Dx. According to information of CS extension, we classified patients with codes 0-400 as mild extension and those with codes 440-800 as grievous extension. All patients were inconsistently separated into two groups (training group, *n* = 4,837, and validation group, *n* = 2,419).

## 3. Methods

Intergroup comparisons were analyzed using Pearson's chi-squared test. An odds ratio (OR) and a 95% confidence interval (CI) were evaluated by a single factor and a multivariate logistic regression model. Variables with significant differences in univariate analysis were included in the logistic regression model for multivariate analysis. With the multivariate analysis results as the basis, by means of R 3.4.1 software (Institute for Statistics and Mathematics, Vienna, Austria; http://www.r-project.org/), a nomogram was constructed. Statistical analyses were performed with IBM SPSS statistics trial ver. 25.0 (IBM, Armonk, NY, USA). All reported *p* values lower than 0.05 were considered significant.

## 4. Results

### 4.1. Patients Characteristics

7,256 target patients were contained in our study. Patients were randomly distributed into training cohort and validation cohort in the ratio of 2 : 1 for building the metastatic predictive model. The parameter of patients in the training and validation cohorts are listed in Table 1. The percentages of patients with metastatic GIST in the training cohort and the validation cohort were 17.12% (828/4837) and 17.49% (423/2419), respectively. The majority of the patients were elder (>55 years) and male. White people comprised 68.22% of the study population. More than half of patients were married (57.35%) and purchased health insurance (76.34%). Tumor location, pathologic grade, tumor size, lymph nodes, extension, and mitotic count were significantly different between nonmetastasis and metastasis for both of training and validation groups.

### 4.2. Establishment of Metastatic Nomogram

Univariate analysis of variables with significant differences were included in the logistic regression model for multivariate analysis. The independent GIST metastatic odds ratios (ORs) for gender, insurance, tumor location, pathologic grade, tumor size, lymph nodes, extension, and mitotic count were presented in Table 2 for the logistic model. Metastatic status of GIST was highly associated with most characteristics in this study, including tumor location, pathologic grade, tumor size, lymph nodes, extension, and mitotic count.

**Figure 1:**
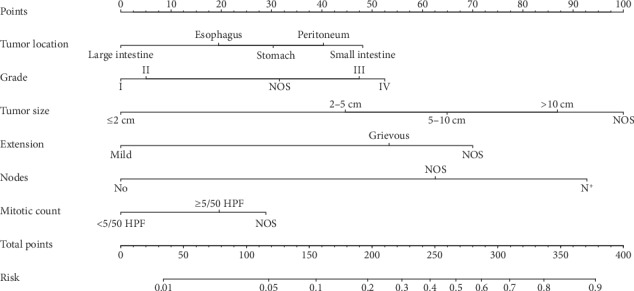
Nomograms for predicting the synchronous metastatic GISTs.

The significantly independent risk factors identified by multivariate analyses were integrated to construct the nomogram for predicting synchronous metastatic diseases. The point scale at the top of each nomograms was used first to give every risk variable a score; then, the scale at the bottom of each nomogram was used (adding up the scores of all variables) to predict synchronous mGIST rates. The nomogram discrimination for synchronous mGIST prediction revealed that tumor size contributed most to metastasis, followed by lymph nodes, extension, pathologic grade, tumor location, and mitotic count (Figure 1).

### 4.3. Verification of Metastatic Nomogram

**Figure 2:**
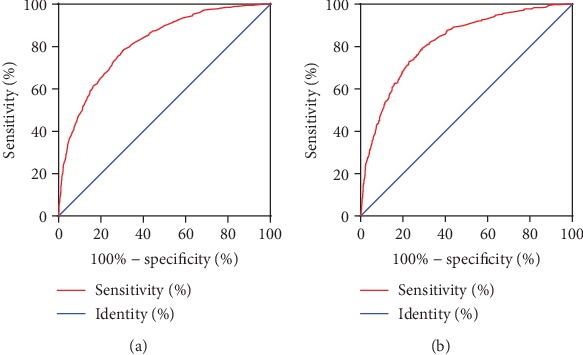
Area under the ROC of the nomogram in (a) the primary cohort and (b) the validation cohort.

**Figure 3:**
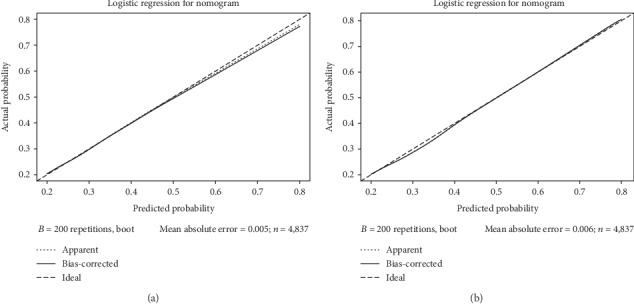
The calibration curves for predictions of synchronous metastatic GISTs (a) in the training cohort and (b) in the validation cohort.

Internal validation for nomograms was performed by *C*-index, Brier score, receiver operating characteristic (ROC) curve, and calibration. *C*-index values of predictions were 0.821 (95% CI: 0.805-0.836) and 0.815 (95% CI: 0.800-0.831), and Brier score were 0.109 and 0.112 in training and validation groups, respectively (Table 3). Both of them suggest that these models made accurate predictions. The values of area under the ROCs were 0.813 (*p* < 0.001, Figure 2(a)) in the primary cohort and 0.819 (*p* < 0.001, Figure 2(b)) in the validation cohort, showing outstanding sensitivity and specificity of the nomogram. Through the calibration curves (Figures 3(a) and 3(b)), nomogram prediction proved to have excellent agreement with actual metastatic diseases.

## 5. Discussion

The results of this study show that 1 out of 6 patients with GISTs presents with synchronous metastases. Considering such a high metastatic ratio, we have created an effective and accurate nomogram, which is related to cumulative risk score of synchronous mGIST, based on tumor and demographic variables available at the time of diagnosis that could be incorporated into clinical practice to guide surveillance and management strategies of GISTs.

Gaitanidis et al. also explored the synchronous metastatic risk factors of GISTs based on the SEER database [7], but their research had some shortcomings. First of all, their research was limited to liver metastases. Secondly, their research did not construct a nomogram, which can guide clinical practices better. This study was dedicated to building a comprehensive scoring system and made up for these shortcomings. Meanwhile, most studies estimated the metastatic risk of GIST based on tumor location, tumor size, and mitotic count but ignored some other features of tumor [2, 7, 8]. Our nomogram showed that small intestine held the highest metastatic risk. Moreover, synchronous metastatic risk was positively correlated with tumor size and mitotic counts in patients with GISTs. These results were consistent with most of previous studies.

It is now well accepted that all GISTs can exhibit malignant behavior and none can be labeled to be definitely benign based on clinicopathologic features. Lymphatic metastasis and tumor extension are peculiar features of malignant tumors. In fact, Gaitanidis et al. proved that lymph node metastasis was associated with mGIST [9]. Pathological grade was also associated with recurrence and metastasis in many other tumors more than the GIST [10–12]. This study believed that regional lymph node, grade, and extension should not be passed over owing to the significant meaning in the logistic regression model. In addition, these clinical features may have a certain relevance with special types of GISTs. For example, regional lymph nodes, but not the mitotic counts, can be used as a metastatic risk factor in patients with SDH- (succinate dehydrogenase-) deficient GISTs [13]. Therefore, we incorporated these clinical features into the nomogram. Surprisingly, the parameters of grade, regional lymph nodes, and extension presented higher risk scores than tumor location and mitotic counts. In addition, this study contained some demographic variables without statistical significance unfortunately, such as age, sex, and race.

Some clinicians lack a knowledge base to assess metastatic risk of GISTs since they are a rare tumor type. And it is the reason why part of the data source is NOS in patients with GISTs. This study suggested that NOS should not be ignored and was included in the predictive nomogram. The various guidelines consistently suggested that tumor location, tumor size, and mitotic counts need to be evaluated in patients with GISTs [14–17]. Interestingly, NOS hold the highest risk score in these recommended indicators, but median risk score regarding grade and lymph nodes. It fully reflected the increased metastatic risk caused by some inexperienced clinicians who ignored the progress of diagnosis and treatment of GISTs. Therefore, the nomogram is equally applicable to referral patients, even if the previous visit information may not be perfect.

To our best knowledge, it is the first study to excavate the metastatic risk score of GISTs based on the SEER database. However, there were also some limitations existing in our study. First, this study was a kind of retrospective study which might have selection bias because not all the GIST patients had routine tests for metastasis resulting in an underestimated incidence of metastasis. Second, the SEER database did not provide such information about the status of KIT, DOG1, and PDGFRA, which are the important immunohistochemical markers for GIST diagnosis and prognosis. Finally, external validation is still required to verify the validity of this nomogram.

## 6. Conclusion

This study created and examined a new nomogram that can evaluate synchronous metastatic diseases in patients with GISTs, and these brand-new predicted methods could be incorporated into clinical practice to guide surveillance and management strategies in GISTs.

## Figures and Tables

**Table 1:** Characteristics of patients in the training and validation groups.

Characteristic	Training group (*n* = 4,837)			Validation group (*n* = 2,419)		
	Nonmetastasis (*n* = 4,009)	Metastasis (*n* = 828)	*p*	Nonmetastasis (*n* = 1,996)	Metastasis (*n* = 423)	*p*
Gender			<0.001			<0.001
Female	1,965 (49.01%)	342 (41.30%)		979 (49.05%)	166 (39.24%)	
Male	2,044 (50.99%)	486 (58.70%)		1,017 (50.95%)	257 (60.76%)	
Age (years)			0.322			0.919
≤40	227 (5.66%)	57 (6.88%)		125 (6.26%)	27 (6.38%)	
41-55	870 (21.70%)	196 (23.67%)		431 (21.59%)	89 (21.04%)	
56-70	1,584 (39.51%)	292 (35.27%)		754 (37.78%)	165 (39.01%)	
>70	1,328 (33.13%)	283 (34.18%)		686 (34.37%)	142 (33.57%)	
Race			0.057			0.475
White	2,701 (67.37%)	586 (70.77%)		1,373 (68.79%)	290 (68.56%)	
Black	751 (18.73%)	141 (17.03%)		345 (17.28%)	85 (20.09%)	
Others	526 (13.12%)	97 (11.71%)		264 (13.23%)	47 (11.11%)	
NOS	31 (0.77%)	4 (0.48%)		14 (0.70%)	1 (0.24%)	
Insurance			<0.001			0.312
Yes	3,107 (77.50%)	586 (70.77%)		1,533 (76.80%)	313 (74.00%)	
No	116 (2.89%)	33 (3.99%)		56 (2.81%)	17 (4.02%)	
NOS	786 (19.61%)	209 (25.24%)		407 (20.39%)	93 (21.99%)	
Marital status			0.917			0.922
Married	2,306 (57.52%)	465 (56.16%)		1,148 (57.52%)	242 (57.21%)	
Unmarried	1,484 (37.02%)	331 (39.98%)		749 (37.53%)	160 (37.83%)	
NOS	219 (5.46%)	32 (3.86%)		99 (4.96%)	21 (4.96%)	
Tumor location			0.009			0.032
Large intestine	216 (5.39%)	35 (4.23%)		94 (4.71%)	11 (2.60%)	
Peritoneum	78 (1.95%)	34 (4.11%)		47 (2.35%)	18 (4.26%)	
Stomach	2,599 (64.83%)	468 (56.52%)		1,306 (65.43%)	253 (59.81%)	
Small intestine	1,093 (27.26%)	289 (34.90%)		535 (26.80%)	139 (32.86%)	
Esophagus	23 (0.57%)	2 (0.24%)		14 (0.70%)	2 (0.47%)	
Pathologic grade			<0.001			<0.001
I	641 (15.99%)	35 (4.23%)		307 (15.38%)	13 (3.07%)	
II	451 (11.25%)	43 (5.19%)		214 (10.72%)	25 (5.91%)	
III	147 (3.67%)	56 (6.76%)		76 (3.81%)	19 (4.49%)	
IV	195 (4.86%)	78 (9.42%)		87 (4.36%)	45 (10.64%)	
NOS	2,575 (64.23%)	616 (74.40%)		1,312 (65.73%)	321 (75.89%)	
Tumor size (cm)			<0.001			<0.001
≤2	495 (12.35%)	16 (1.93%)		221 (11.07%)	9 (2.13%)	
2-5	1,230 (30.68%)	109 (13.16%)		644 (32.26%)	48 (11.35%)	
5-10	1,241 (30.96%)	223 (26.93%)		629 (31.51%)	108 (25.53%)	
>10	833 (20.78%)	307 (37.08%)		389 (19.49%)	154 (36.41%)	
NOS	210 (5.24%)	173 (20.89%)		113 (5.66%)	104 (24.59%)	
Lymph nodes			<0.001			<0.001
Negative	3,783 (94.36%)	548 (66.18%)		1,884 (94.39%)	306 (72.34%)	
Positive	94 (2.34%)	123 (14.86%)		62 (3.11%)	49 (11.58%)	
NOS	132 (3.29%)	157 (18.96%)		50 (2.51%)	68 (16.08%)	
Extension			<0.001			<0.001
Mild	3,051 (76.10%)	280 (33.82%)		1,565 (78.41%)	145 (34.28%)	
Grievous	726 (18.11%)	346 (41.79%)		347 (17.38%)	156 (36.88%)	
NOS	232 (5.79%)	202 (24.40%)		84 (4.21%)	122 (28.84%)	
Mitotic count			<0.001			<0.001
≤5/50 HPF	1,513 (37.74%)	130 (15.70%)		750 (37.58%)	69 (16.31%)	
>5/50 HPF	1,615 (40.28%)	432 (52.17%)		788 (39.48%)	202 (47.75%)	
NOS	881 (21.98%)	266 (32.13%)		458 (22.95%)	152 (35.93%)	

NOS: not otherwise specified.

**Table 2:** Adjusted odds ratio (OR) with 95% confidence interval (CI) and *p* value for selected tumor and demographic characteristics as indicators of metastatic diseases among GIST patients.

Characteristics	OR	95% CI	*p* value
Gender			0.124
Female	Reference		1.000
Male	1.146	0.963-1.363	0.124
Insurance			0.094
Yes	Reference		1.000
No	1.475	0.944-2.305	0.088
NOS	0.878	0.706-1.092	0.244
Tumor location			0.001
Large intestine	Reference		1.000
Peritoneum	2.185	1.156-4.131	0.016
Stomach	1.823	1.163-2.855	0.009
Small intestine	2.564	1.607-4.092	<0.001
Esophagus	1.445	0.293-7.120	0.651
Pathologic grade			<0.001
I	Reference		1.000
II	1.071	0.646-1.774	0.791
III	2.518	1.495-4.241	0.001
IV	2.798	1.721-4.551	<0.001
NOS	1.859	1.247-2.771	0.002
Tumor size (cm)			<0.001
≤2	Reference		1.000
2-5	2.366	1.353-4.138	0.003
5-10	3.516	2.040-6.060	<0.001
>10	5.370	3.110-9.272	<0.001
NOS	7.124	4.005-12.671	<0.001
Lymph nodes			<0.001
Negative	Reference		1.000
Positive	6.153	4.531-8.355	<0.001
NOS	3.991	3.035-5.249	<0.001
Extension			<0.001
Mild	Reference		1.000
Grievous	2.886	2.368-3.518	<0.001
NOS	3.991	3.035-5.249	<0.001
Mitotic count			<0.001
≤5/50 HPF	Reference		1.000
>5/50 HPF	1.554	1.207-2.000	0.001
NOS	1.788	1.348-2.372	<0.001

NOS: not otherwise specified.

**Table 3:** Model performance following internal validation.

	Training group	Validation group
*C*-index	0.821 (0.805-0.836)	0.815 (0.800-0.831)
Brier score	0.109	0.112

## Data Availability

These data were derived from the Surveillance, Epidemiology and End Results (SEER) database (https://seer.cancer.gov/) and identified using the SEER^∗^Stat software (version 8.3.5) (https://seer.cancer.gov/seerstat/).
